# Carpal Tunnel Syndrome: The Role of Occupational Factors Among 906 Workers

**DOI:** 10.5812/traumamon.6554

**Published:** 2012-07-31

**Authors:** Mohammad Ghasemi, Maryam Rezaee, Farzaneh Chavoshi, Mohammad Mojtahed, Ehsan Shams Koushki

**Affiliations:** 1 Health Research Center, Baqiyatallah University of Medical Sciences, Tehran, IR Iran; 2 Trauma Research Center, Baqiyatallah University of Medical Sciences, Tehran, IR Iran; 3 Occupational Disease Research Center, Tehran University of Medical Sciences, Tehran, IR Iran; 4 Department of Otolaryngology, Imam Khomeini Hospital, Tehran University of Medical Sciences, Tehran, IR Iran

**Keywords:** Carpal Tunnel Syndrome, Occupational Medicine, Workload, Risk Factor

## Abstract

**Background:**

Carpal tunnel syndrome (CTS) is common in the industrial setting. However,there is a controversy about the sole role of occupational ergonomic hazards in CTS.

**Objectives:**

This study was conducted among assembling workers of a detergent factory and computer users with the aims of A) determination of CTS prevalence and B) evaluation of personal risk factors and level of exposure to occupational risk factors via Quick Exposure Check (QEC).

**Materials and Methods:**

In this descriptive cross-sectional study, 906 cases (332 assembling workers and 574 computer workers) were enrolled. CTS was assessed by symptoms on the Katz hand diagram and physical examination. QEC technique was applied to evaluate physical exposure to the risk factors.

**Results:**

According to this study, the prevalence of probable CTS was 14% in men and 8.9% in women; the rate of probable CTS was significantly higher in assembly workers than in computer users (*P* < 0.001). Mean age and work duration in the probable CTS group was statistically higher than in non-CTS group. But both groups were in the same range (fewer than 30, *P* = 0.024, 0.004); BMI in the probable CTS group was slightly lower than in non CTS group, but BMI in both groups were in the normal range. Wrist ratio > 0.7 correlated with increased risk of probable CTS (*P* < 0.001) Prevalence of probable CTS was significantly higher in third and fourth levels of QEC (*P* < 0.001).

**Conclusions:**

Although this article had limitations, our findings suggest that the level of occupational exposure is an indicator of CTS development.

## 1. Background

Carpal Tunnel Syndrome (CTS) is considered as the most common entrapment neuropathy and repetitive trauma disorder (1). Prevalence rates of CTS are reported as 3.0-5.8% among females and 0.6-2.1 % among males (2, 3). Although literature is in favor of the association between ergonomic hazards and CTS, evidence regarding this relationship is unclear (1). On the other hand, each occupational ergonomic hazard alone does not result in CTS and probably combination of several factors is needed (1). Working tasks involving high pressure, high force, repetition and segmental vibration are associated with higher rates of CTS (4). Repetition of wrist flexion and extension is the greatest and the most accepted occupational risk factor. Frequency of the task and the percentage at the time spent on the repetitive task are important (4). Moreover, repetitive wrist movements lasting more than 30 seconds and working more than 50% of total work time in repetitive movement patterns are considered significant (4-6). Occupational exposures do not explain the incidence of CTS, and it is believed that other factors may be involved, such as personal (intrinsic and social) factors, unsafe actions and unhealthy lifestyles (7, 8). It has been shown that only 8.29% of CTS cases are due to occupational risk factors and other variables such as BMI, age, and wrist depth to width ratio are more important (9). The roles of cigarette smoking, alcohol abuse and excessive use of caffeine have been noted in some studies (10).

## 2. Objectives

The aim of this study was to assess the prevalence of CTS and evaluation of personal and ergonomic risk factors based on QEC among assembly workers and computer operators.

## 3. Materials and Methods

In this cross-sectional study, participants were selected from hand-involved occupations in Tehran, Iran, from August 2008 to May 2009. The participants were recruited from a large company which manufactured detergents such as liquid soap, shampoo, dish washing liquid, etc. We selected computer operators and assembling workers among these participants. All workers with more than two years of employment at the plant who worked at least 40 hours per week and were at least 18 years of age were included in our study. Workers with a history of certain disorders such as rheumatoid arthritis, diabetes mellitus, cervical radiculopathy, hypothyroidism, thoracic outlet syndrome, trauma to the upper limbs and medically diagnosed CTS prior to starting the current occupation were excluded. The Ethics Committee of Tehran University of Medical Sciences approved the study protocol, and all participants filled out informed consent forms.

Data collection was carried out using the questionnaires, medical history and examination. Overall, 1213 workers were invited. Two hundred and forty five workers were excluded because they met the exclusion criteria. Finally, 906 (332 assembling workers and 574 computer operators) entered the study. All subjects were asked to complete a self-administrated demographic questionnaire, including age, sex, employment duration, smoking habit, having a regular exercise program and the dominant hand. The diagnosis of probable CTS was based on: 1. (pain, paraesthesia or decreased sensitivity present in the thumb, index or middle finger on the Katz hand diagram, and 2. A positive Tinel test, compression test with a combination of Phalen’s test, decreased sensation in median nerve distribution or weakness of thumb abduction or wasting of the thenar eminence (11). We used a Quick Exposure Check (QEC) for better job analysis. Completing Katz hand diagram and job analysis by the QEC method were done by a trained occupational hygienist. We measured BMI and wrist index in all subjects and then recorded the results in their questionnaires.

Job’s analysis was carried out to assess the work ergonomic determinants. In this way, we used the QEC method as an observational analysis method of occupational task movement to determine occupational risk factors affecting the musculoskeletal system. For each anatomic area (including neck, shoulder-arm and wrist-hand), assessment was performed separately using a total of 16 questions with two, three or four optional answers (12). In this way, we assessed some ergonomic risk factors within the wrist-hand area, exposure to segmental vibration, work speed and occupational stress. We evaluated awkward wrist/hand posture (wrist flexion/extension, side bending (ulnar/radial deviation), rotation of the wrist around the axis of the forearm and wrist/hand movement excluding the movement with the fingers. In phase 2, the score for each question was entered into a matrix to calculate the combined effect of risk factors. Finally, the score of wrist/hand area was converted to an exposure level (low, moderate, high and very high) according to score ranges defined for wrist/hand level. We employed chi-square test and independent sample t-test for categorical and continuous data, respectively. Because our independent variables such as age, level of exposure and duration of employment had been high co-linearity, we could not use logistic regression analysis (13). Data analysis was performed with SPSS 16.

## 4. Results

The mean age of the workers was 27.82 years with standard deviation of 5.98; 56.4% were males and 43.3% were females. Mean employment duration was 8.25 ± 3.68. Among these workers, 332 were assembling workers (36.6%) and 574 were computer workers (63.4%); 43.82% of workers had regular exercise programs and 20% of them had a history of cigarette smoking. Mean body mass index (BMI) and wrist index were 25.11 ± 3.81 and 0.67 ± 0.09, respectively. Based on CTS criteria, 107 persons (11.8%) had probable CTS which was in the dominant hand in 93 cases (86.9%). Table 1 and Table 2 show physical work-load factors and demographic characteristics between the probable CTS group and the non-CTS group.

**Table 1:** Comparison of Demographic Characteristics Between Probable CTS Group and the non-CTS Group

	Number	Probable or Possible CTS a group, Mean ± SD	Non-CTS group, Mean ± SD	*P *value
Age	906	29.14±6.44	27.64±5.9	0.024
Sex				0.022
Male	514	72 ± 14	442 ± 86	
Female	392	35 ± 8.9	357 ± 91.1	
Duration of Employment	906	9.94 ± 6.44	8.03 ± 5.54	0.004
Smoking				0.095
Yes	181	28 ± 15.5	153 ± 84.5	
No	725	79 ± 10.9	646 ± 89.1	
High	240	41 ± 17.1	199 ± 82.9	
Exercise				< 0.001
Yes	509	20 ± 3.9	489 ± 96.1	
No	397	87 ± 21.9	310 ± 78.1	
Occupation				< 0.001
Assembling worker	332	62 ± 18.7	270±81.3	
Computer worker	574	45 ± 7.8	529 ± 92.2	
BMI a	906	24.11 ± 4.18	25.25 ± 3.74	0.008
Wrist index				< 0.001
≤ 0.7	696	69 ± 9.9	627 ± 90.1	
> 0.7	210	38 ± 18.1	172 ± 81.9	

^a^Abbreviations: BMI: Body Mass Index; CTS: Carpal Tunnel Syndrome

**Table 2:** Comparison of Exposure Level of Physical Work Load Risk Factors Between Possible or Probable CTS Groups and the Non-CTS Group

	Number	Probable or Possible CTS Group, Mean ± SD	Non-CTS Group, Mean ± SD	*P *value
Wrist Exposure level				< 0.001
No	725	79 ± 10.9	646 ± 89.1	
Low risk	181	3 ± 1.7	178 ± 98.3	
Moderate	123	2 ± 1.6	121 ± 98.4	
High	482	64 ± 13.3	418 ± 86.7	
Very High	120	38 ± 31.7	82 ± 68.3	
Segmental vibration				0.005
Low	666	66 ± 9.9	600 ± 90.1	
High	240	41 ± 17.1	199 ± 82.9	
Pace				0.005
Low	392	41 ± 10.5	351 ± 89.5	
Moderate	304	28 ± 9.2	276 ± 90.8	
High	210	38 ± 18.1	172 ± 81.9	
Stress				<0.001
Low risk	245	1 ± 0.4	244 ± 99.6	
Moderate	331	58 ± 17.5	273 ± 82.5	
High	210	18 ± 8.6	192 ± 91.4	
Very High	120	30 ± 25	90 ± 75	

## 5. Discussion

Our findings showed that the prevalence of probable CTS was 14% in men and 8.9% in women. The prevalence of CTS in our study was higher than general population (1-5% in the general population and 6-15% in the industrial setting). This is similar to the prevalence rate found in previous studies (2, 3, 14-18). In this study, mean age of the patients was slightly higher in the probable CTS group. In most literature reviews, the prevalence of CTS development was found to be increased in patients 40-60 years-old (19). It is likely that this difference is not clinically important in our study. Previous studies have found women to be at higher risk for CTS (7, 19, 20). It is likely that the greater number of men involved in physically demanding work (21-24) place men are at higher risk than women in industrial settings. The difference in smoking was not statistically significant between the two groups. According to some studies, smoking is not more prevalent in CTS patients (21-25). There was a negative association between regular exercise and probable CTS (*P* < 0.001). Different studies have shown that exercise helps the CTS symptoms subside. Exercise may help hand tendons in the wrist move more easily, preventing swelling or inflammation of tendons in the carpal tunnel (26, 27). In our study, probable CTS was more prevalent in assembly workers than in computer operators. According to some reports, the risk for CTS in computer operators is still much lower than occupations involving heavy labor. Although more than 10% of the computer operators complained of CTS symptoms, however, evidence shows that association between CTS and computer use is weak (28, 29).

This difference can be due to the higher prevalence of repetitive movements of the wrist or hand, forceful hand grip and handling of loads in assembly jobs. According to some studies, BMI is a systemic condition associated with the development of CTS (30-33). Our findings showed that BMI in the probable CTS group was slightly lower than the non CTS group. Although this difference was significant, but it is not clinically important, because BMI is in the normal range in both groups in the current study. We found that a wrist ratio > 0.7 correlated with increased risk in probable CTS which was consistent with the results of the previous studies (21, 30-34). The role of the wrist ratio in CTS development is not fully understood, but several explanations have been proposed. In the squarer shaped wrist, median nerve compression may increase during flexion and extension movements of the digits and wrist. Thus, anatomy of the wrist may predispose individuals to the carpal tunnel syndrome. Table 2 shows the highest prevalence of probable CTS is in the very high exposure level of the wrist. This is in agreement with the findings from other researchers (35, 36). Our results demonstrated an association between QEC risk level and the prevalence rate of probable CTS which implied that QEC was an appropriate system for determining the level of exposure to work-related CTS risks (37). High levels of segmental vibration contribute to the higher prevalence of probable CTS (*P* = 0.005).

According to some studies, use of vibrating tools increases the risk of CTS development (38-42). Vibration of hand tools affects blood circulation, and may cause wrist and hand disorders. Patients in the probable CTS group had a significantly high level of pace as compared to patients in the non CTS group (0.005); It seems that when work rate increases, repetition of hand or wrist increases as a result; therefore, the risk of CTS development increases (21, 39). There is a correlation between the stress level and probable CTS. According to some studies, psychological distress is predictive of CTS (43-45). Some studies did not find any association between CTS and psychosocial risk factors (46).

Although this article had limitations, our findings suggest that level of occupational exposure is an indicator of CTS development. Since most studies have found a relationship between CTS and psychosocial risk factors, it is likely that these factors be implicated in the development of CTS. Previous studies indicate that women are at higher risk for CTS than men; It is likely that the greater number of men involved in physically demanding work places them at higher risk to develop CTS than women in industrial settings. The prevalence of CTS in our study was higher than found in the general population which is similar to the prevalence rate found in previous studies. Associations between CTS and workplace activities have been established which could be due to repeated movements of the wrist. Ergonomic exercises to prevent CTS can be appropriate, and control of occupational risk factors in the workplace can aid rehabilitation of the affected worker.

### 5.1. Limitations

A limitation in this study was its cross-sectional nature. Analytic studies can better confirm the relationship between the exposure and outcome. Another limitation was that no inquiry was made regarding previous jobs; however, according to some studies, only exposure in the recent job was associated with CTS. In this study, we did not employ nerve conduction studies as a gold standard test for CTS diagnosis. The combination of symptoms based on Katz hand diagram, physical examination and nerve conduction studies can result in more accurate diagnosis. We used the QEC method for evaluation of physical risk factors in the workplace. The tool not only focuses on physical workplace factors but also evaluates psychosocial factors. Tasks can normally be assessed within 10 minutes. It has a scoring system and exposure levels implicated as a guide for intervention. Although this method has several advantages, the validity of exposure scores for different action levels is questionable.

## References

[1] Aroori S, Spence RA (2008). Carpal tunnel syndrome.. Ulster Med J.

[2] Atroshi I, Gummesson C, Johnsson R, Ornstein E, Ranstam J, Rosen I (1999). Prevalence of carpal tunnel syndrome in a general population.. JAMA.

[3] de Krom MC, Knipschild PG, Kester AD, Thijs CT, Boekkooi PF, Spaans F (1992). Carpal tunnel syndrome: prevalence in the general population.. J Clin Epidemiol.

[4] Silverstein BA, Fine LJ, Armstrong TJ (1987). Occupational factors and carpal tunnel syndrome.. Am J Ind Med.

[5] Ham SJ, Kolkman WF, Heeres J, den Boer JA (1996). Changes in the carpal tunnel due to action of the flexor tendons: visualization with magnetic resonance imaging.. J Hand Surg Am.

[6] Werner R, Armstrong TJ, Bir C, Aylard MK (1997). Intracarpal canal pressures: the role of finger, hand, wrist and forearm position.. Clin Biomech (Bristol, Avon)..

[7] Becker J, Nora DB, Gomes I, Stringari FF, Seitensus R, Panosso JS (2002). An evaluation of gender, obesity, age and diabetes mellitus as risk factors for carpal tunnel syndrome.. Clin Neurophysiol.

[8] Lam N, Thurston A (1998). Association of obesity, gender, age and occupation with carpal tunnel syndrome.. Aust N Z J Surg.

[9] Gerr F, Letz R (1992). Risk factors for carpal tunnel syndrome in industry: blaming the victim?. J Occup Med.

[10] Rempel D, Bach JM, Gordon L, So Y (1998). Effects of forearm pronation/supination on carpal tunnel pressure.. J Hand Surg Am.

[11] Sluiter JK, Rest KM, Frings-Dresen MH (2001). Criteria document for evaluating the work-relatedness of upper-extremity musculoskeletal disorders.. Scand J Work Environ Health.

[12] BPNSea LG, Stanton N (2005). Quick Exposure Checklist for the Assessment of Workplace Risks for Work-Related Musculoskeletal Disorders (WMSDs).. Handbook of Human Factors and Ergonomics Methods.

[13] Agresti A (2012). Categorical Data Analysis.

[14] Einhorn N, Leddy JP (1996). Pitfalls of endoscopic carpal tunnel release.. Orthop Clin North Am.

[15] Ferry S, Pritchard T, Keenan J, Croft P, Silman AJ (1998). Estimating the prevalence of delayed median nerve conduction in the general population.. Br J Rheumatol.

[16] Prick JJ, Blaauw G, Vredeveld JW, Oosterloo SJ (2003). Results of carpal tunnel release.. Eur J Neurol.

[17] Stevens JC, Sun S, Beard CM, O’Fallon WM, Kurland LT (1988). Carpal tunnel syndrome in Rochester, Minnesota, 1961 to 1980.. Neurology.

[18] Tanaka S, Wild DK, Seligman PJ, Behrens V, Cameron L, Putz-Anderson V (1994). The US prevalence of self-reported carpal tunnel syndrome: 1988 National Health Interview Survey data.. Am J Public Health.

[19] Phalen GS (1970). Reflections on 21 years’ experience with the carpal-tunnel syndrome.. JAMA.

[20] Ferry S, Hannaford P, Warskyj M, Lewis M, Croft P (2000). Carpal tunnel syndrome: a nested case-control study of risk factors in women.. Am J Epidemiol.

[21] Armstrong T, Dale AM, Franzblau A, Evanoff BA (2008). Risk factors for carpal tunnel syndrome and median neuropathy in a working population.. J Occup Environ Med.

[22] Giersiepen K, Eberle A, Pohlabeln H (2000). Gender differences in carpal tunnel syndrome? occupational and non-occupational risk factors in a population-based case-control study.. Ann Epidemiol.

[23] McDiarmid M, Oliver M, Ruser J, Gucer P (2000). Male and female rate differences in carpal tunnel syndrome injuries: personal attributes or job tasks?. Environ Res.

[24] Nathan PA, Keniston RC, Myers LD, Meadows KD, Lockwood RS (1998). Natural history of median nerve sensory conduction in industry: relationship to symptoms and carpal tunnel syndrome in 558 hands over 11 years.. Muscle Nerve.

[25] Karpitskaya Y, Novak CB, Mackinnon SE (2002). Prevalence of smoking, obesity, diabetes mellitus, and thyroid disease in patients with carpal tunnel syndrome.. Ann Plast Surg.

[26] Nathan PA, Wilcox A, Emerick PS, Meadows KD, McCormack AL (2001). Effects of an aerobic exercise program on median nerve conduction and symptoms associated with carpal tunnel syndrome.. J Occup Environ Med.

[27] Thomas RE, Butterfield RK, Hool JN, Herrick RT (1993). Effects of exercise on carpal tunnel syndrome symptoms.. Appl Ergon.

[28] Palmer KT, Harris EC, Coggon D (2007). Carpal tunnel syndrome and its relation to occupation: a systematic literature review.. Occup Med (Lond)..

[29] Thomsen JF, Gerr F, Atroshi I (2008). Carpal tunnel syndrome and the use of computer mouse and keyboard: a systematic review.. BMC Musculoskelet Disord.

[30] Boz C, Ozmenoglu M, Altunayoglu V, Velioglu S, Alioglu Z (2004). Individual risk factors for carpal tunnel syndrome: an evaluation of body mass index, wrist index and hand anthropometric measurements.. Clin Neurol Neurosurg.

[31] Kouyoumdjian JA, Zanetta DM, Morita MP (2002). Evaluation of age, body mass index, and wrist index as risk factors for carpal tunnel syndrome severity.. Muscle Nerve.

[32] Moghtaderi A, Izadi S, Sharafadinzadeh N (2005). An evaluation of gender, body mass index, wrist circumference and wrist ratio as independent risk factors for carpal tunnel syndrome.. Acta Neurol Scand.

[33] Sharifi-Mollayousefi A, Yazdchi-Marandi M, Ayramlou H, Heidari P, Salavati A, Zarrintan S (2008). Assessment of body mass index and hand anthropometric measurements as independent risk factors for carpal tunnel syndrome.. Folia Morphol (Warsz)..

[34] Galea LA, Gatt R, Sciberras C (2007). Hand and wrist configurations in patients with Carpal Tunnel Syndrome.. Malta Med J.

[35] Choobineh A, Tabatabaei SH, Mokhtarzadeh A, Salehi M (2007). Musculoskeletal problems among workers of an Iranian rubber factory.. J Occup Health.

[36] Mirmohamadi M, Seraji JN, Shahtaheri J, Lahmi M, Ghasemkhani M (2004). Evaluation of Risk Factors Causing Musculoskeletal Disorders Using QEC Method in a Furniture Producing Unite.. Iran J Publ Health.

[37] David G, Woods V, Li G, Buckle P (2008). The development of the Quick Exposure Check (QEC) for assessing exposure to risk factors for work-related musculoskeletal disorders.. Appl Ergon.

[38] Barcenilla A, March LM, Chen JS, Sambrook PN (2012). Carpal tunnel syndrome and its relationship to occupation: a meta-analysis.. Rheumatology (Oxford)..

[39] Burke FD, Lawson IJ, McGeoch KL, Miles JN, Proud G (2005). Carpal tunnel syndrome in association with hand-arm vibration syndrome: a review of claimants seeking compensation in the Mining Industry.. J Hand Surg Br.

[40] Hagberg M, Morgenstern H, Kelsh M (1992). Impact of occupations and job tasks on the prevalence of carpal tunnel syndrome.. Scand J Work Environ Health.

[41] Shiri R, Miranda H, Heliovaara M, Viikari-Juntura E (2009). Physical work load factors and carpal tunnel syndrome: a population-based study.. Occup Environ Med.

[42] Wieslander G, Norback D, Gothe CJ, Juhlin L (1989). Carpal tunnel syndrome (CTS) and exposure to vibration, repetitive wrist movements, and heavy manual work: a case-referent study.. Br J Ind Med.

[43] Leclerc A, Franchi P, Cristofari MF, Delemotte B, Mereau P, Teyssier-Cotte C (1998). Carpal tunnel syndrome and work organisation in repetitive work: a cross sectional study in France. Study Group on Repetitive Work.. Occup Environ Med.

[44] Leclerc A, Landre MF, Chastang JF, Niedhammer I, Roquelaure Y (2001). Upper-limb disorders in repetitive work.. Scand J Work Environ Health.

[45] Roquelaure Y, Mariel J, Dano C, Fanello S, Penneau-Fontbonne D (2001). Prevalence, incidence and risk factors of carpal tunnel syndrome in a large footwear factory.. Int J Occup Med Environ Health.

[46] van Rijn RM, Huisstede BM, Koes BW, Burdorf A (2009). Associations between work-related factors and the carpal tunnel syndrome--a systematic review.. Scand J Work Environ Health.

